# Application of provincial data in mathematical modelling to inform sub-national tuberculosis program decision-making in South Africa

**DOI:** 10.1371/journal.pone.0209320

**Published:** 2019-01-25

**Authors:** Piotr Hippner, Tom Sumner, Rein MGJ Houben, Vicky Cardenas, Anna Vassall, Fiammetta Bozzani, Don Mudzengi, Lindiwe Mvusi, Gavin Churchyard, Richard G. White

**Affiliations:** 1 The Aurum Institute, Johannesburg, South Africa; 2 TB Modelling Group, TB Centre, The London School of Hygiene and Tropical Medicine, London, United Kingdom; 3 Department of Infectious Disease Epidemiology, The London School of Hygiene and Tropical Medicine, London, United Kingdom; 4 Department of Global Health and Development, The London School of Hygiene and Tropical Medicine, London, United Kingdom; 5 TB Control and Management, National Department of Health, Pretoria, South Africa; 6 School of Public Health, University of the Witwatersrand, Johannesburg, South Africa; 7 Advancing Care & Treatment (ACT) for TB/HIV, South African Medical Research Council, Johannesburg, South Africa; Medical Research Council, SOUTH AFRICA

## Abstract

South Africa has the highest tuberculosis (TB) disease incidence rate in the world, and TB is the leading infectious cause of death. Decisions on, and funding for, TB prevention and care policies are decentralised to the provincial governments and therefore, tools to inform policy need to operate at this level. We describe the use of a mathematical model planning tool at provincial level in a high HIV and TB burden country, to estimate the impact on TB burden of achieving the 90-(90)-90 targets of the Stop TB Partnership Global Plan to End TB. “TIME Impact” is a freely available, user-friendly TB modelling tool. In collaboration with provincial TB programme staff, and the South African National TB Programme, models for three (of nine) provinces were calibrated to TB notifications, incidence, and screening data. Reported levels of TB programme activities were used as baseline inputs into the models, which were used to estimate the impact of scale-up of interventions focusing on screening, linkage to care and treatment success. All baseline models predicted a trend of decreasing TB incidence and mortality, consistent with recent data from South Africa. The projected impacts of the interventions differed by province and were greatly influenced by assumed current coverage levels. The absence of provincial TB burden estimates and uncertainty in current activity coverage levels were key data gaps. A user-friendly modelling tool allows TB burden and intervention impact projection at the sub-national level. Key sub-national data gaps should be addressed to improve the quality of sub-national model predictions.

## Introduction

South Africa has the highest tuberculosis (TB) incidence rate in the world [1], and the disease is the leading cause of death [2]. The estimated burden of disease differs significantly between provinces with the rate of microbiologically confirmed TB (in 2012) ranging from approximately 400/100,000/year in Limpopo Province to almost 1,200/100,000/year in KwaZulu-Natal [3]. In addition, the drivers of the epidemic also differ between provinces due to variation in factors including HIV prevalence, levels of drug resistance, and the capacity of the health system to identify and treat TB [4, 5].

Recent modelling studies have shown that scaling up existing interventions in all of South Africa has the potential to greatly reduce TB incidence and mortality, and to be cost-effective [6–8]. However, the vast majority of modelling studies have focussed on the national level and have not addressed potential differences between provinces [6, 7, 9–12].

Evidence-based TB prevention and treatment strategies are agreed upon nationally and implemented by the provinces. The allocation of budgets to specific activities also takes place primarily at the provincial level. Provincial TB programmes are resource limited and often have to make decisions without information on the likely impact of different intervention options in their setting. With the introduction of additional ring-fenced TB funding (TB conditional grant) in 2017 [13], the availability of an additional sub-national planning tool to project impact is highly desirable.

In this article we describe the application of TIME (Tuberculosis Impact Module and Estimates), a user friendly TB transmission model at sub-national level in South Africa. We used the model to estimate the impact on TB incidence and mortality from 2017 to 2035 of scale-up of TB screening, linkage and retention interventions to achieve the 90-(90)-90 TB targets of the Stop TB Partnership Global Plan to End TB [14]. This work was conducted as part of the South African National TB Think Tank [15].

## Methods

### TIME model

This work used the TIME module of the Spectrum suite (Version 5∙54 Beta 3) [16, 17]. The TIME model is a user friendly, age-structured, dynamic, compartmental transmission model of TB. The model includes three disease states: susceptible, latent infection, and active disease. The model is stratified by TB treatment history, smear status, multidrug resistant TB (MDR-TB) status and HIV. Smear-negative cases are assumed to be less infectious than those with smear-positive TB by a factor of 0.22 [18]. The MDR-TB strata represents those with multi-drug resistant and rifampicin resistant TB, consistent with the reporting of these cases by WHO as a single group. The HIV positive population is stratified by CD4 count and time on ART. ART is assumed to reduce the risk of developing TB and the risk of HIV and TB associated mortality. As a component of Spectrum, TIME Impact automatically incorporates country data on TB (notifications, incidence estimates, mortality estimates etc.) from the Global TB Programme (GTB) at the World Health Organization, HIV estimates (HIV incidence and prevalence, ART coverage, ART eligibility thresholds) from the Aids Impact Model (AIM, a module of Spectrum) and demographic projections from DemProj (the demographic module in Spectrum). A full description of the TIME model can be found in the supplementary material of Houben et al.[16]

TIME can be applied at sub-national level using local data. For this work, each province in South Africa was considered a separate unit assuming no mixing or migration between provinces. The models used provincial level AIM and demographic files supplied by UNAIDS.

Use of the model requires the user to provide input data reflecting current levels of TB control, including rates of screening, treatment outcomes and sensitivity and specificity of the diagnostic algorithm. Where possible we used values based on data reported and collated by the provincial TB programmes. These are described in more detail below.

### Province selection

TB burden varies across each of the nine provinces of South Africa and is influenced by numerous demographic and epidemiological factors. KwaZulu-Natal (KZN), Limpopo (LP) and Western Cape (WC) provinces were selected for this modelling study to illustrate variation in key epidemiological factors, population size, TB burden, HIV prevalence, ART coverage and, TB treatment success (Table 1).

**Table 1:** Demographic and epidemiological characteristics of the selected provinces. Classifications of “High”, “Medium”, “Low” are relative to national values for South Africa.

Indicator (year)	KwaZulu-Natal (KZN)	Limpopo (LP)	Western Cape (WC)
**Population size (2015)**	Large 10,919,100	Medium 5,726,800	Medium 6,200,100
**TB notification rate (2015)**	High 671/100,000	Low 297/100,000	High 687/100,000
**Total MDR-TB notifications (2015)**	High 3,763 (34/100,000)	Low 490 (8.6/100,000)	Medium 1,750 (28/100,000)
**HIV prevalence (2016)**	High 18%	Low 8%	Low 7%
**ART coverage (2015)**	High 55%	Medium 46%	Medium 48%
**Drug-sensitive TB treatment success [as proxy for programme performance] (2014)**	Below average (falling) 76.8%	Below average (increasing) 64.9%	Above average (stable) 81.9%

### Data sources

#### Burden estimates

Provincial notification data were obtained from national electronic TB register (ETR.net) reports. Provincial TB incidence was estimated using national TB notification data and the national TB incidence estimate from the WHO TB global report to derive a national case detection ratio (CDR), the proportion of incident TB cases that are notified to the TB programme [1]. In the absence of provincial-level CDR estimates, the national CDR was applied to the provincial notification data (assuming the CDR was the same in all provinces) to obtain an estimate of provincial TB incidence. While this is an obvious simplification, we chose to use this approach rather than introduce other assumptions into the model. TB mortality was derived using national vital registration (VR) data in combination with WHO mortality estimates to calculate the percentage of estimated deaths captured by VR systems [1, 19]. This percentage was applied to the province VR data to obtain approximate provincial mortality estimates [19].

#### Programme performance

TB treatment outcomes data were obtained from national electronic TB register (ETR.net) reports. TB screening and testing data was derived from district health information system (DHIS) quarterly reports. Nationally, pre-treatment initial loss to follow-up (ILTFU, the proportion of those diagnosed with TB who did not start treatment) has been estimated at 17% [20, 21]. The Health Systems Trust (HST) estimates of loss to follow-up were available at provincial level [19]. However, these were defined as the proportion of new smear-positive patients who did not start treatment or whose treatment was interrupted for two or more months. To derive province level estimates for pre-treatment ILTFU among all TB cases we used the ratio of the provincial to national HST estimates to adjust the national ILTFU estimate of 17%. The baseline levels of these TB programmatic activities are shown in Table 2.

**Table 2:** Summary of modelled intervention scenarios showing baseline coverage and target values, by province. KZN = KwaZulu-Natal, LP = Limpopo and WC = Western Cape.

			KZN	LP	WC
Intervention 1: Clinic visiting headcount screened for TB (%)		Baseline (2015)	24∙4	70	10∙4
		Intervention (2021)	90	90	90
Intervention 2: ILTFU (%)		Baseline (2015)	12∙5	14∙2	26∙9
		Intervention (2021)	2∙5	2∙8	5∙4
Intervention 3:	DS-TB treatment success (%)	Baseline (2015)	76∙8	64∙9	81∙9
		Intervention (2021)	85	85	85
	DR-TB treatment success (%)	Baseline (2015)*	50	50	50
		Intervention (2021)	67	67	67

*DR-TB treatment success is assumed to be the same in each province and is based on national data.

While there is considerable uncertainty in the province level data, in particular screening numbers, we aimed to use data as reported by the provinces where possible. This allowed us to explore which input data were the key drivers of model projections and where efforts should be focussed on improving collection and reporting of data to allow better model estimates of intervention impact. To minimise the effect of misreporting of screening data we used data from subsequent steps in the diagnostic cascade (numbers of patients with symptoms, numbers tested for TB, etc.) to estimate province level values for the specificity and linkage to testing post screening.

Three regional modelling workshops took place in June 2016 which were attended by members of provincial TB departments. These workshops built on two previously held national workshops in which the TIME modelling tool was showcased to the National TB Programme (NTP). In the regional workshops, draft versions of the provincial models were presented and attendees were provided the opportunity to work directly with the TIME model and discuss the available data and model assumptions. While the workshops did not follow a formal elicitation approach (e.g. Delphi Process) province TB data managers provided input and expert opinion on the quality, accuracy and representativeness of their local data, and identified important data gaps to address in the future. The draft models were updated based on these discussions.

### Baseline scenario

The baseline model scenario represented the historic, current and projected burden assuming no future increase in TB and HIV care in South Africa with the exception of antiretroviral therapy (ART) coverage which was assumed to increase to 90% of all known HIV infected individuals by 2017 in line with the UNAIDS 90-90-90 targets. Historical values for programmatic TB parameters were based on the earliest year for which routine data were available and we assumed that all existing activities continue at their current levels until 2035. The current value of TB treatment success was calculated as the average of the last five year’s data to account for large annual fluctuations. Screening, diagnosis, and initiation of treatment were not modelled explicitly but were simplified based on the diagnostic algorithm from the National TB Treatment guidelines [22]. Full details of the assumptions and parameter values can be found in the S1 File.

### Model calibration

Each provincial model was fitted to the number of notified TB cases, estimated TB incidence for the years 2002 to 2015 [1], and notified MDR-TB cases from 2013 to 2015 (Personal Communication, Provincial TB managers, March 2016). There has been a large increase in TB screening in the last few years so the models were also fitted to TB screening data from 2013 to 2015 (Personal Communication, Provincial TB managers, March 2016). The model was manually calibrated by adjusting the screening rate, the effective contact rate and the relative rate of presentation of healthy individuals for screening (compared to those with TB).

### Interventions

**Fig 1:**
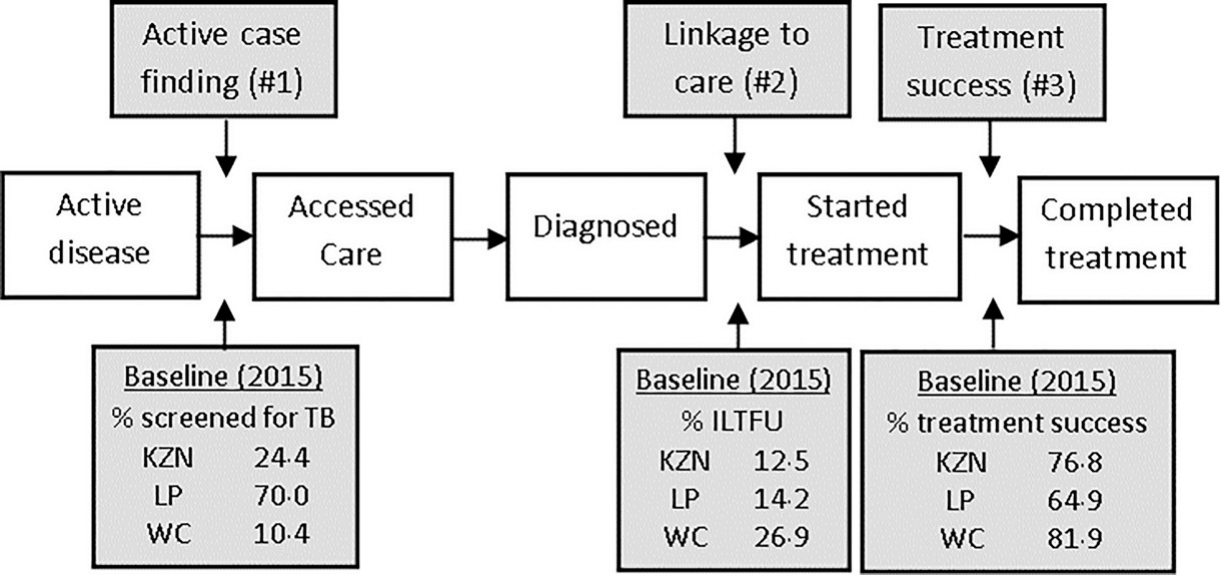
TB care pathway. The key steps in the care pathway (from active disease to successful treatment) are represented by the white boxes. Grey boxes indicate the steps on the pathway targeted by each of the interventions and the baseline values used in the model. KZN = KwaZulu-Natal, LP = Limpopo and WC = Western Cape.

In line with the 90-(90)-90 targets from the Stop TB Partnership’s Global Plan to End TB [14], three TB prevention and care interventions were modelled as part of this exercise. Fig 1 shows the TB care pathway indicating which part of the pathway the interventions target as well as the baseline values for the key indicator of each of the interventions. The primary aim of these interventions was to find TB cases (intervention #1), link them to care (intervention #2) and ensure successful treatment (intervention #3).

Intervention #1 modelled an increase in the proportion of primary health clinic attendees screened for TB. We assumed screening was based on cough triage, a shortened version of the WHO screening algorithm consisting of a single question which asks patients whether they have a cough for more than two weeks (or of any duration if they are known to be HIV-positive). Intervention #2 modelled an improved linkage to care for those diagnosed with TB. We assumed ILTFU was reduced by 80% by 2021 compared to baseline values. Intervention #3 assumed increased treatment success rates for drug-susceptible TB (to 85%) and drug-resistant TB (to 67%), based on the standard six and 24 month regimens respectively.

Linear scale-up from 2016 to 2021 was assumed for all interventions. The target values for scale-up were determined following detailed discussions with representatives of the NTP and scientific experts (Table 2). All interventions were assumed to remain at their target levels from 2021 to 2035.

## Results

### Engagement with sub-national TB programme staff

A series of three regional workshops were held which involved the provincial TB managers and their teams as part of the sub-national modelling process. Attendees gained hands-on experience of using the modelling planning tool and the workshop activities illustrated the relationship between quality data as model inputs and how variability can greatly influence the outputs and predicted impact.

### Baseline fit and projection

**Fig 2:**
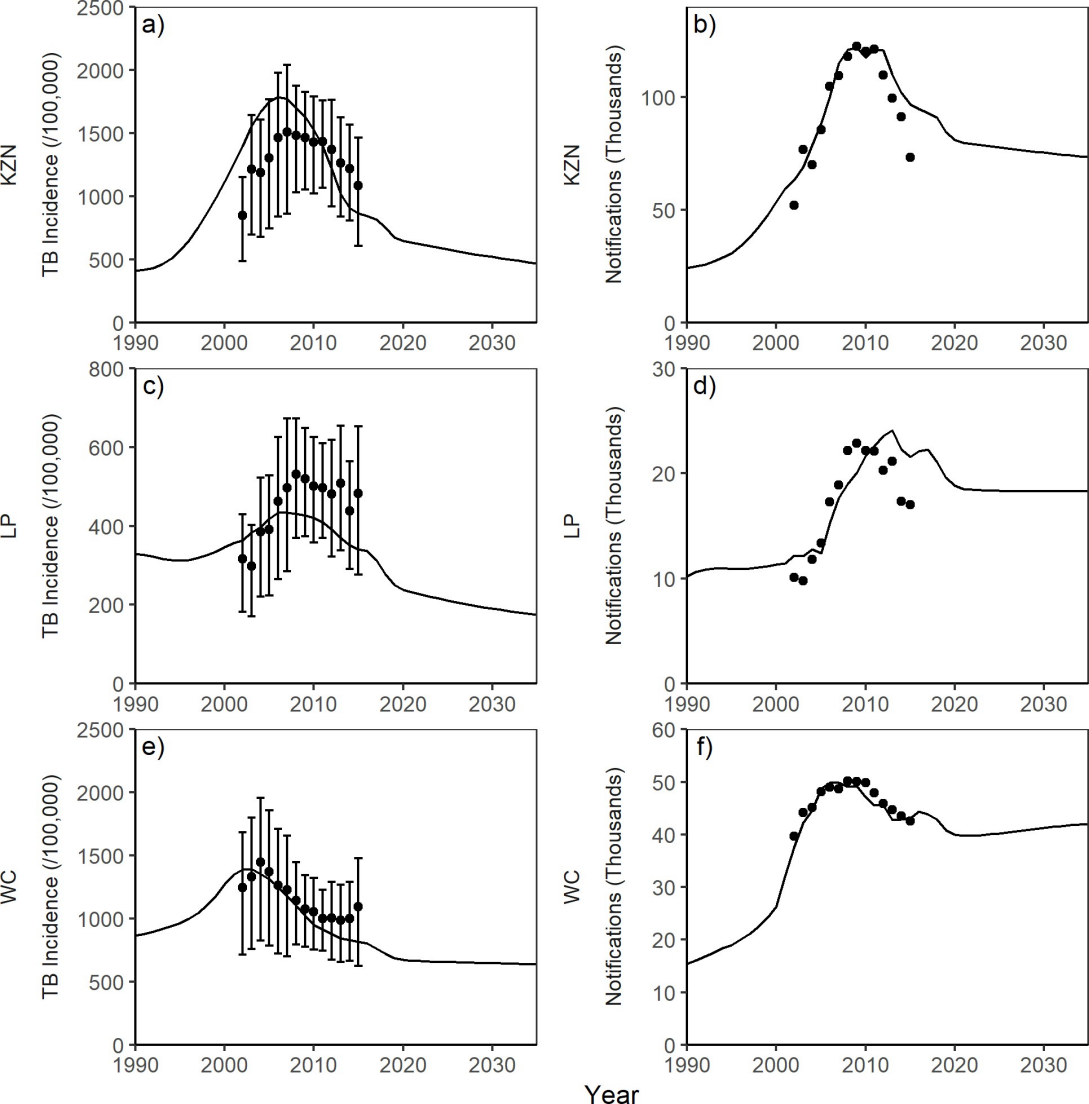
Baseline plots of TB notifications (number) and TB incidence (/100,000) for each province. KZN = KwaZulu-Natal, LP = Limpopo and WC = Western Cape. (Markers = provincial estimates derived from WHO data with plausible range; line = model).

Fig 2 shows the baseline fit of TB notifications and incidence for the three provincial models. The model outputs match the observed trends in TB notifications in all three provinces (Fig 2B, 2D and 2F). The TB incidence model outputs fall within the uncertainty range of the estimated incidence for all three provinces (Fig 2A, 2C and 2E). The models suggest that the incidence peaked in 2006 for KwaZulu-Natal (Fig 2A) and Limpopo (Fig 2C) while in Western Cape the peak was earlier in 2003 (Fig 2E).

Following their peaks, all three models show a trend of decreasing incidence until 2019 followed by gradual decline until 2035, although the decline in Western Cape is smaller than those predicted for KwaZulu-Natal and Limpopo. Under the baseline scenario from 2015 to 2035, the models suggest that the TB incidence will decrease by 46%, 49% and 22% for KwaZulu-Natal, Limpopo, and Western Cape respectively (Fig 2A, 2C and 2E). Similarly, the TB mortality is predicted to decrease by 58%, 63% and 26% for KwaZulu-Natal, Limpopo, and Western Cape respectively (Fig B in S1 File).

### Intervention impact

**Fig 3:**
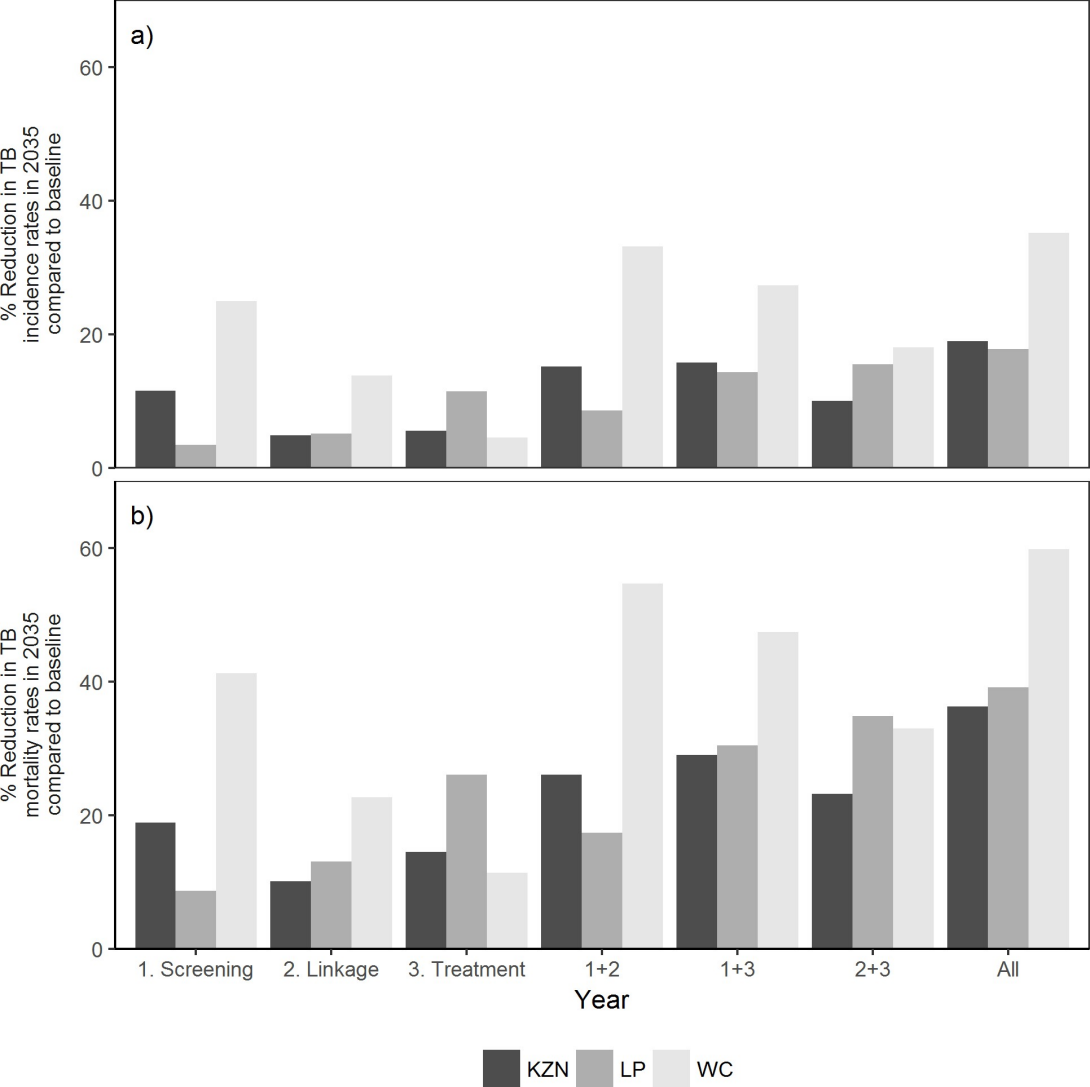
Projected percentage reduction in incidence and mortality rate in 2035, compared to the baseline scenario, by province and intervention. KZN = KwaZulu-Natal, LP = Limpopo and WC = Western Cape.

Fig 3 shows the percentage reduction in the incidence and mortality rates in 2035 compared to baseline for each of the intervention scenarios. As expected, the combination of all three interventions was predicted to result in the largest reductions in TB incidence and mortality in all three provinces (Fig 3A and 3B, rightmost bars).

In KwaZulu-Natal, Fig 3A shows that increased TB screening (intervention #1) was projected to have the greatest impact on incidence with an 11∙5% reduction with improved linkage into care (interventions #2) and increased treatment success (intervention #3) having less than half the impact (4∙9% and 5∙6% respectively).

A similar pattern was observed in Western Cape where intervention #1 had the biggest impact on incidence with a reduction of 25.0%, while interventions #2 and #3 showed smaller reductions (13∙8% and 4∙6% respectively).

In contrast, in Limpopo improved treatment success (intervention #3) was projected to have the largest reduction in incidence (11∙5%) compared to intervention #2 (5∙2%) and intervention #1 (3∙4%) (Fig 3A).

The impact of the interventions on TB mortality showed similar patterns to those seen for the impact on incidence, although the percentage impact was greater for all interventions in all three provinces (Fig 3B).

## Discussion

All models predicted a continuing trend of decreasing TB incidence and mortality, consistent with the recent observed decrease in TB notifications in South Africa. The projected impacts of the interventions differed by province and appear to be mainly driven by differences in current reported coverage levels rather than differences in the underlying epidemiology. The absence of sub-national TB burden estimates and uncertainty in current activity coverage levels are important data gaps that should be addressed at the provincial level.

The regional workshops and involvement of the provincial TB managers and their teams were critical components of the sub-national modelling process. They provided an opportunity for staff from the provincial TB programmes to have first-hand experience of the modelling planning tool and offered insights into the workings and requirements of the modelling process. This fostered an understanding of the importance of good quality data and how changes in model inputs can greatly influence the outputs and predicted impact of what is being modelled. The process also highlighted to programme staff areas where strengthening data collection could improve future decision making.

The TIME model has been used previously to assess the TB epidemic in South Africa [6–8], but this was the first instance of the model being used at the sub-national level in South Africa. The previous work [6] suggested that at a national level, the greatest impact could be achieved by scaling up screening of primary health clinic attendees. In contrast, our sub-national analysis suggests screening would only be the most effective strategy in 2 of the 3 provinces studied. The contrasting impact of the interventions in the provinces reflects that the TB epidemic in South Africa differs by province and that the responses need to take into account the provincial epidemiological context. They also highlight an important role for the use of sub-national planning tools.

Several studies have used modelling to look at the TB epidemic in South Africa and assess the impact of TB prevention and care interventions [6, 9–12]. These studies used different models to assess the impact of different aspects of TB prevention and care interventions covering various time periods for their predictions, ranging from ten to 38 years. However, none of these analyses aimed to inform policy at the subnational level. Several modelling studies have looked at the TB epidemic at sub-national level in South Africa [23, 24]. Gilbert *et al*. [23] looked at the impact of combinations of TB interventions over a ten-year period but the analysis was confined to the rural setting of Msinga sub-district in KwaZulu-Natal. Another group modelled the TB epidemic in the urban setting of the city of Cape Town in Western Cape which was underpinned by access to high quality TB notification and programmatic data [24]. While these models were able to characterise the epidemic in a single setting they did not compare the effect of interventions across provinces using the same modelling framework.

This analysis suggested that the scale-up of key interventions had the potential to significantly reduce TB incidence and mortality in all three provinces. The impact of the interventions was greatly affected by the baseline levels of the core activities of each intervention. Increasing coverage of TB screening (intervention #1) was predicted to have a substantial impact on incidence and mortality in Western Cape and KwaZulu-Natal compared to a very modest effect in Limpopo as the reported baseline coverage of Limpopo’s TB screening was already high and close to the target value. Achieving the 90% screening target in Limpopo required an increase of 29% in coverage levels compared to Western Cape where the TB screening coverage had to increase almost nine-fold in order to reach target levels. This suggested that very different levels of resources may be required to achieve the targets in different settings.

Several of the key parameters used in the models were not available at the provincial level and had to be calculated from various data sources which were of variable quality and completeness. This included national estimates of incidence and mortality which were themselves based on modelled data. The use of assumptions to derive provincial-level data resulted in estimates with great uncertainty which might not have reflected the reality on the ground. Certain estimates were derived assuming that parameter values were the same across provinces which may have masked the heterogeneity in the epidemiology and intervention coverage at provincial level. The greater scrutiny that was applied to TB data allowed gaps in quality and reporting to be highlighted. The routine programmatic data used to parameterise the models may have been of variable quality and completeness which varied by province. The models did not provide granular control over the TB care pathway and algorithms, resulting in crude estimates of what is in reality a complex process.

The TIME model includes the effect of HIV on TB but does not currently include other known risk factors for TB such as diabetes or smoking. Strategies to detect and prevent TB in these risk groups may form an important part of future efforts to control TB in South Africa. Future work could consider differences in the prevalence of diabetes and other risk factors between provinces when assessing the likely impact of strategies focussed on these risks groups. The scale-up of interventions illustrated the possible impact but did not provide specific details of how they could be implemented. These models were not meant to offer definitive estimates of future disease burden or intervention impact but instead to illustrate the effect of prioritising different components of TB prevention and care. In addition, we did not consider the resource or financial implications of scaling up these interventions in each province. Analysis of the costs and cost-effectiveness of different strategies at the province level will provide further evidence on which to base the sub-national planning of TB control activities. This is the focus on ongoing research by our group, incorporating province level impact estimates and differences in service costs at the subnational level.

Ideally, the choice of how best to prioritise TB prevention and care activities within provinces should take into consideration the current levels of TB activities in order to assess where gaps exist. These choices rely on the reporting of high quality and accurate TB data, both on disease burden and programme performance. Incorrect reporting could result in focus being given to activities which may not offer much additional benefit in relation to the resources required to implement them. The use of provincial data and modelling provides insight into the provincial TB epidemics allowing for more attention to be given to the local level context where decisions are best taken on how to tackle the TB epidemic. To ensure the results of these and similar analyses can inform future TB programme efforts it is essential that long-term links are established and maintained between, policy makers, provincial TB programme staff and those generating research evidence. The success of the national TB think tank [15] illustrates the potential for such collaboration.

A user-friendly modelling tool allows burden and intervention impact projection at the sub-national level. Key sub-national data gaps should be addressed to improve the quality of sub-national model predictions.

## Supporting information

S1 File Supporting information. Additional details on model parameterisation and calibration and additional results.
(DOCM)

## References

[1] World Health Organisation. Global Tuberculosis Report. Geneva: 2017.

[2] Statistics South Africa. Mortality and causes of death in South Africa, 2015: Findings from death notification. Pretoria, South Africa2017.

[3] Nanoo A, Izu A, Ismail NA, Ihekweazu C, Abubakar I, Mametja D, et al Nationwide and regional incidence of microbiologically confirmed pulmonary tuberculosis in South Africa, 2004–12: a time series analysis. Lancet Infect Dis. 2015;15(9):1066–76. 10.1016/S1473-3099(15)00147-4 . 26112077

[4] Shisana O, Rehle T, C. SL, Zuma K, Jooste S, N. Z, et al South African National HIV Prevalence, Incidence and Behaviour Survey, 2012 Cape Town, South Africa: Human Sciences Research Council, 2014.

[5] National Institute of Communicable Diseases, editor South African Tuberculosis Drug Resistance Survey 2012–142016; Johannesburg: National Institute for Communicable Diseases.

[6] Houben RMGJ, Menzies NA, Sumner T, Huynh GH, Arinaminpathy N, Goldhaber-Fiebert JD, et al Feasibility of achieving the 2025 WHO global tuberculosis targets in South Africa, China, and India: a combined analysis of 11 mathematical models. The Lancet Global health. 2016;4:e806–e15. 10.1016/S2214-109X(16)30199-1 . 27720688 PMC6375908

[7] Menzies NA, Gomez GB, Bozzani F, Chatterjee S, Foster N, Baena IG, et al Cost-effectiveness and resource implications of aggressive action on tuberculosis in China, India, and South Africa: a combined analysis of nine models. Lancet Glob Health. 2016 Epub 2016/10/11. 10.1016/s2214-109x(16)30265-0 . 27720689 PMC5527122

[8] National Department of Health, South African National AIDS Council, editors. South African HIV and TB Investment Case—Reference Report Phase 12016; Pretoria: Department of Health, South Africa, and South African National AIDS Council.

[9] Dowdy DW, Chaisson Re Fau—Maartens G, Maartens G Fau—Corbett EL, Corbett El Fau—Dorman SE, Dorman SE, Proc Natl Acad Sci USA. Impact of enhanced tuberculosis diagnosis in South Africa: a mathematical model of expanded culture and drug susceptibility testing. 1998. D—NLM: PMC2516234 EDAT- 2008/08/13 09:00 MHDA- 2008/09/17 09:00 CRDT- 2008/08/13 09:00 AID—0800965105 [pii] AID - 10.1073/pnas.0800965105 PST—ppublish. PMC2516234 18695217

[10] Pretorius C, Menzies Na Fau—Chindelevitch L, Chindelevitch L Fau—Cohen T, Cohen T Fau—Cori A, Cori A Fau—Eaton JW, Eaton Jw Fau—Fraser C, et al. The potential effects of changing HIV treatment policy on tuberculosis outcomes in South Africa: results from three tuberculosis-HIV transmission models. 2014. 10.1097/QAD.0000000000000085 24468944

[11] Dye C, Glaziou P Fau—Floyd K, Floyd K Fau—Raviglione M, Raviglione M, Annu Rev Public H. Prospects for tuberculosis elimination. 2012. 10.1146/annurev-publhealth-031912-114431 23244049

[12] Knight GM, Dodd PJ, Grant AD, Fielding KL, Churchyard GJ, White RG. Tuberculosis Prevention in South Africa. PLOS ONE. 2015;10:e0122514 10.1371/journal.pone.0122514 25849558 PMC4388715

[13] Treasury National. Estimates of National Expenditure 2017. Pretoria, South Africa: 2017.

[14] Stop TB Partnership. The Paradigm Shift 2016–2020. Geneva, Switzerland: 2016.

[15] White RG, Charalambous S, Cardenas V, Hippner P, Sumner T, Bozzani F, et al Evidence informed policymaking at the country level: lessons learned from the South African Tuberculosis Think Tank. under review, IJTLD. 2017. 10.5588/ijtld.17.0485 PMC5947421 29862943

[16] Houben RM, Lalli M, Sumner T, Hamilton M, Pedrazzoli D, Bonsu F, et al TIME Impact—a new user-friendly tuberculosis (TB) model to inform TB policy decisions. BMC Med. 2016;14:56 Epub 2016/03/26. 10.1186/s12916-016-0608-4 . 27012808 PMC4806495

[17] Avenir Health. Spectrum Glastonbury, CT: Avenir Health; 2017 Available from: http://www.avenirhealth.org/software-spectrum.php.

[18] Behr MA, Warren SA, Salamon H, Hopewell PC, Ponce de Leon A, Daley CL, et al Transmission of Mycobacterium tuberculosis from patients smear-negative for acid-fast bacilli. Lancet. 1999;353(9151):444–9. Epub 1999/02/16. . 9989714 10.1016/s0140-6736(98)03406-0

[19] Health Systems Trust. Health Indicators. 2016.

[20] Churchyard GJ, Stevens WS, Mametja LD, McCarthy KM, Chihota V, Nicol MP, et al Xpert MTB/RIF versus sputum microscopy as the initial diagnostic test for tuberculosis: a cluster-randomised trial embedded in South African roll-out of Xpert MTB/RIF. 2015 10.1016/S2214-109X(15)00100-X 26187490

[21] MacPherson P, Houben RM, Glynn JR, Corbett EL, Kranzer K, Bull World Health O. Pre-treatment loss to follow-up in tuberculosis patients in low- and lower-middle-income countries and high-burden countries: a systematic review and meta-analysis. 2014 D—NLM: PMC3949536 EDAT- 2014/03/14 06:00 MHDA- 2015/07/03 06:00 CRDT- 2014/03/14 06:00 PHST- 2013/05/20 [received] PHST- 2013/10/02 [revised] PHST- 2013/10/03 [accepted] AID—10.2471/BLT.13.124800 [AID—BLT.13.124800 [pii] PST—ppublish. 10.2471/BLT.13.124800 24623906 PMC3949536

[22] National Department of Health. National Tuberculosis Management Guidelines 2014. Pretoria, South Africa: 2014.

[23] Gilbert JA, Long EF, Brooks RP, Friedland GH, Moll AP, Townsend JP, et al Integrating Community-Based Interventions to Reverse the Convergent TB/HIV Epidemics in Rural South Africa. PLOS ONE. 2015;10:e0126267 10.1371/journal.pone.0126267 25938501 PMC4418809

[24] Blaser N, Zahnd C, Hermans S, Salazar-Vizcaya L, Estill J, Morrow C, et al Tuberculosis in Cape Town: An age-structured transmission model. Epidemics. 2016;14:54–61. 10.1016/j.epidem.2015.10.001. 26972514 PMC4791535

