# Supplementary material for: Application of provincial data in mathematical modelling to inform sub-national tuberculosis program decision-making in South Africa
Source: PLoS One. 2019 Jan 25;14(1):e0209320. doi: 10.1371/journal.pone.0209320 (PMC6347133; doi:10.1371/journal.pone.0209320)
Supplement: S1 File — Additional details on model parameterisation and calibration and additional results. (DOCM) [file pone.0209320.s001.docm]

**Application of provincial data in mathematical modelling to inform sub-national tuberculosis program decision-making in South Africa**

**Authors:** Piotr Hippner, Tom Sumner, Rein MGJ Houben, Vicky Cardenas, Anna Vassall, Fiammetta M Bozzani, Don Mudzengi, Lindiwe Mvusi, Gavin Churchyard, Richard G White

**Supporting Information**

**S.1. Baseline scenario**

The following sections provide more detail on the assumptions made in the baseline scenario.

**S.1.1. Tuberculosis Screening**

South Africa has only recently introduced a standardised indicator set for TB screening. Historical data for the rates of screening were collected by the provinces using their own tools and methodology.

We assume that the rates of passive screening (of those with or without TB) are independent of HIV status but do depend on TB status. Rates of passive screening are also assumed to be lower for individuals with smear-negative TB (by a factor of 0.8) compared to those with smear-positive TB. The rates of passive screening (in those with and without TB) are estimated by fitting the model (see below). Passive screening is assumed to be based on presence of any symptom with a sensitivity and specificity of 84 (95% CI: 76–93) and 74 (95% CI: 53–95) respectively.

Increases in active TB screening in recent years (2013 to 2015) were included in the baseline models by increasing the screening rates (in those with and without TB) to match to reported numbers of screens conducted in each province (Personal Communication, Provincial TB managers, March 2016).

**S.1.2. Diagnostic algorithm**

Individuals identified as having possible TB will enter one of 2 diagnostic pathways defined by the initial test: smear microscopy or gene Xpert. In each year, we assume some proportion of the population will receive smear or Xpert as initial test (see table S1). Figure A shows a simplified representation of the algorithm based on the SA guidelines [[1](#_ENREF_1)].

People identified with TB symptoms

% SSM

% GX

SSM +

SSM -

GX +

GX -

Treat

Follow up

Follow up +

Follow up -

Treat

Treat

Follow up

Follow up +

Follow up -

Treat

**Figure A** - Simplified representation of the SA diagnostic algorithm

**S.1.3. Sensitivity and specificity of diagnostic algorithms**

As the TIME model does not explicitly include the stages of the diagnostic algorithm, a net sensitivity and specificity were calculated for each arm of the algorithm (smear or Xpert as initial test). The overall sensitivity or specificity in a given year was calculated as the weighted average by initial test (see table A).

The net sensitivity of each algorithm is defined by:

$$P\left( A\cap B\cap C \right)=P\left( A \right) \times P\left( B \right) \times P(C)$$

Where A = sensitivity of first screening tool, B = sensitivity of second screening tool and C = sensitivity of confirmatory test.

The net specificity of each algorithm is defined by:

$$P\left( A\cup B\cup C \right)= P\left( A \right)+P\left( B \right)+P\left( C \right)- P\left( A\cap B \right)-P\left( A\cap C \right)-P\left( B\cap C \right)+P(A\cap B\cap C)$$

The sensitivity decreases as more tools are added to the algorithm; however, the specificity (and therefore the PPV) will increase.

**Smear as initial test**

**Smear positive TB cases**

Algorithm: Symptom screen (any TB symptom) followed by 2x sputum microscopy

Sensitivity:

Sensitivity of symptom screen = 0.84 [[2](#_ENREF_2)]

Sensitivity of sputum microscopy = 1 (assumed by definition)

Algorithm sensitivity = 0.84*1 = 0.84

Specificity:

Specificity of symptom screen = 0.74 [[2](#_ENREF_2)]

Specificity of sputum microscopy = 0.98

Algorithm specificity = 0.74 + 0.98 – (0.74*0.98)

**Smear negative TB cases**

Algorithm: Symptom screen (any TB symptom) followed by 2x sputum microscopy followed by antibiotics followed by x-ray/clinical investigation (assume sputum will be negative by definition)

Sensitivity:

Sensitivity of symptom screen = 0.84 [[2](#_ENREF_2)]

Sensitivity of xray/clinical diagnosis following antibiotic trial = 0.24 [[2](#_ENREF_2)]

Algorithm sensitivity = 0.84*0.24 = 0.2016

Specificity:

Specificity of symptom screen = 0.74 [[2](#_ENREF_2)]

Specificity of xray/clinical diagnosis following antibiotic trial = 0.94

Algorithm specificity = 0.74 + 0.94 – (0.74*0.94) = 0.9844

**Xpert as initial test** **(does not account for Xpert negative algorithm in HIV+)**

**Smear positive TB cases**

Algorithm: Symptom screen followed by Xpert

Sensitivity:

Sensitivity of symptom screen = 0.84 [[2](#_ENREF_2)]

Sensitivity of Xpert = 0.98 [[3](#_ENREF_3)]

Algorithm sensitivity = 0.84*0.98 = 0.8232

Specificity:

Specificity of symptom screen = 0.74 [[2](#_ENREF_2)]

Specificity of sputum microscopy = 0.99 [[3](#_ENREF_3)]

Algorithm specificity = 0.74+0.99 – (0.74*0.99) = 0.9974

**Smear negative TB cases**

Algorithm: Symptom screen followed by Xpert

Sensitivity:

Sensitivity of symptom screen = 0.84 [[2](#_ENREF_2)]

Sensitivity of Xpert = 0.68 [[3](#_ENREF_3)]

Algorithm sensitivity = 0.84*0.68 = 0.5712

Specificity:

As for smear positive cases

In HIV+ individuals estimates of sensitivity for smear-negative TB are much lower at around 47% [[4](#_ENREF_4)]. So we assume sensitivity of the Xpert algorithm in HIV+, smear negative cases is 0.84*0.47 = 0.3948.

| **Year** | **Utilisation of Xpert as first line diagnostic (%)** |
| --- | --- |
| 2010 | 0.00 |
| 2011 | 0.22 |
| 2012 | 0.43 |
| 2013 | 0.65 |
| 2014 | 0.73 |
| 2015 | 0.80 |
| 2016 | 0.80 |

**Table A.** **Utilisation of Xpert as first line diagnostic test.** The assumed coverage is 65% in 2013, 80% in 2015 and linear scale up in between.

**S.1.4. Initial loss to follow up (ILTFU)**

Information on ILTFU, the gap between positive diagnosis and treatment initiation is limited. Based on a systematic review published in 2014 [[5](#_ENREF_5)] and data from the Xtend study [[6](#_ENREF_6)] we assumed that 17% of those with a positive diagnosis did not initiate treatment.

The Health Systems Trust (HST), reports data for loss to follow-up (amongst new smear positive patients) both nationally at provincial level. This is defined as the proportion on patients who did not start treatment or whose treatment was interrupted for two consecutive months. To estimate the ILTFU for all TB cases at a provincial level we calculated the ratio of the provincial: national HST values and used this to scale the national ILTFU estimate of 17% for each of the three provinces. The result values are shown in table B. These were used in the model to parameterise the proportion of diagnosed cases who are linked into care (100 – ILFTU).

| **Year** | **ILTFU (%)** | | |
| --- | --- | --- | --- |
|  | **KZN** | **LP** | **WC** |
| 2001 | 17.1 | 14.5 | 17.3 |
| 2002 | 21.9 | 9.2 | 16.7 |
| 2003 | 25.1 | 11.1 | 16.3 |
| 2004 | 21.6 | 5.9 | 19.6 |
| 2005 | 24.0 | 12.1 | 18.1 |
| 2006 | 23.0 | 15.3 | 18.1 |
| 2007 | 20.2 | 14.8 | 18.4 |
| 2008 | 18.8 | 17.9 | 18.6 |
| 2009 | 17.5 | 18.4 | 17.0 |
| 2010 | 18.0 | 15.0 | 17.0 |
| 2011 | 16.2 | 13.1 | 19.2 |
| 2012 | 13.4 | 13.2 | 21.7 |
| 2013 | 12.0 | 13.2 | 24.3 |

**Table B. Estimated ILTFU for each province**

**S.1.5. Treatment success**

For simplicity, individuals who start treatment are divided into those who are successfully treated (completed + cured) and those who are not. Treatment success was assumed to be independent of HIV status but does depend on drug resistance. Values of treatment success for drug-susceptible cases were taken ETR.net and are shown in table C. To account for short term fluctuations the average of the last five years is used as the value from 2015 onwards. MDR treatment success was assumed to be between 50% for all three provinces across all the years.

| **Year** | **Treatment success (completed + cured) (%)** | | |
| --- | --- | --- | --- |
|  | **KZN** | **LP** | **WC** |
| 2006 | 65.5 | 62.2 | 77.1 |
| 2007 | 63.3 | 63.7 | 79.2 |
| 2008 | 68 | 64.6 | 79.9 |
| 2009 | 70.4 | 65.7 | 80.2 |
| 2010 | 73.7 | 64.5 | 81.6 |
| 2011 | 75.9 | 65.7 | 81.5 |
| 2012 | 78.4 | 64.5 | 81.5 |
| 2013 | 81.8 | 57.6 | 82.6 |
| 2014 | 74 | 72.3 | 82.3 |

**Table C. Treatment success for DS-TB cases, NDOH reports**

**S.2. Model Calibration**

The model for each of the provinces was manually calibrated by adjusting the screening rate, the effective contact rate and the relative rate of presentation of healthy individuals for screening. In addition, several other model parameters were adjusted within their pre-defined ranges in the TIME software to fine-tune the model calibration. The models were matched to reported TB notifications, estimated TB incidence, the reported number of screens and the reported number of MDR treatment initiations.

Table D lists the calibration data and sources used.

Table E gives the parameter values used for each province to achieve the final model fit.

|  | **TB notifications**  **(number)** | | | **TB incidence**  **(per 100,000 population)** | | | **Screened**  **(number)** | | | **MDR treatment initiations**  **(number)** | | |
| --- | --- | --- | --- | --- | --- | --- | --- | --- | --- | --- | --- | --- |
| **Year** | **KZN** | **LP** | **WC** | **KZN** | **LP** | **WC** | **KZN** | **LP** | **WC** | **KZN** | **LP** | **WC** |
| 2002 | 52016 | 10098 | 39650 | 849 | 317 | 1245 | - | - | - | - | - | - |
| 2003 | 76838 | 9797 | 44161 | 1215 | 297 | 1329 | - | - | - | - | - | - |
| 2004 | 69912 | 11832 | 45165 | 1188 | 386 | 1446 | - | - | - | - | - | - |
| 2005 | 85507 | 13366 | 48193 | 1305 | 391 | 1373 | - | - | - | - | - | - |
| 2006 | 104705 | 17301 | 48989 | 1463 | 462 | 1266 | - | - | - | - | - | - |
| 2007 | 109556 | 18910 | 48672 | 1507 | 497 | 1226 | - | - | - | - | - | - |
| 2008 | 118062 | 22189 | 50169 | 1483 | 531 | 1143 | - | - | - | - | - | - |
| 2009 | 122642 | 22836 | 50118 | 1465 | 519 | 1076 | - | - | - | - | - | - |
| 2010 | 120421 | 22138 | 49840 | 1430 | 500 | 1055 | - | - | - | - | - | - |
| 2011 | 121328 | 22121 | 47921 | 1433 | 497 | 1001 | - | - | - | - | - | - |
| 2012 | 109708 | 20272 | 45846 | 1372 | 481 | 1005 | - | - | - | - | - | - |
| 2013 | 99606 | 21151 | 44700 | 1262 | 508 | 985 | 5823050 | 6137835 | - | - | - | - |
| 2014 | 91382 | 17355 | 43563 | 1217 | 437 | 1000 | 8762392 | 7424172 | 475913 | 3754 | 141 | 862 |
| 2015 | 73272 | 17012 | 42565 | 1086 | 483 | 1093 | 9 464 430 | 7941859 | 1156310 | 3965 | 516 | 1758 |

**Table D. Calibration data.** Notifications and Screening numbers were taken from SA NDOH reports and are assumed to represent the total number of screens conducted. Incidence estimates were obtained from WHO estimates of national TB incidence in South Africa and SA NDOH reports.

|  | **KZN** | **LP** | **WC** |
| --- | --- | --- | --- |
| **Effective contact rate (n/year)** | 19.5 | 12.5 | 30 |
| **TB parameters** | | | |
| **HIV-** | | | |
| Develop primary TB following infection (%) | 15 | 15 | 15 |
| Reactivation rate (%/year) | 0.2 | 0.18 | 0.22 |
| Protection provided by prior infection (%) | 65 | 65 | 65 |
| Proportion of cases developing SSpos TB (%) | 40 | 40 | 40 |
| Relative infectiousness of SSneg TB | 0.22 | 0.22 | 0.22 |
| Smear conversion rate (%/year) | 2 | 2 | 2 |
| Self-cure rate (%/year) | 24 | 24 | 24 |
| SSPos TB mortality rate (%/year) | 28 | 28 | 20 |
| SSneg TB mortality rate (%/year) | 8 | 8 | 18 |
| **HIV+ (CD4 > 500)** | | | |
| Develop primary TB following infection (%) | 40.5 | 62.5 | 67 |
| Reactivation rate (%/year) | 0.54 | 0.99 | 1.28 |
| Protection provided by prior infection (%) | 39 | 39 | 39 |
| Proportion of cases developing SSpos TB (%) | 30 | 30 | 30 |
| Relative infectiousness of SSneg TB | 0.1 | 0.1 | 0.1 |
| Smear conversion rate (%/year) | 1.5 | 1.5 | 1.5 |
| Self-cure rate (%/year) | 22.5 | 22.5 | 22.5 |
| SSPos TB mortality rate (%/year) | 81 | 81 | 60 |
| SSneg TB mortality rate (%/year) | 76 | 76 | 40 |
| **MDR Specific Parameters** | | | |
| Relative fitness of MDR strains | 0.76 | 0.9 | 0.79 |
| Rate of acquiring MDR (% per treatment episode) | 1 | 0.5 | 0.6 |
| **ART Specific parameters** | | | |
| **% Reduction in risk of progression to TB** | | | |
| ART < 6m | 15.75 | 15.75 | 15.75 |
| ART 7m-12m | 42.75 | 42.75 | 42.75 |
| ART > 12m | 60 | 70 | 70 |
| **% Reduction in risk of death from TB** | | | |
| ART < 6m | 10.7 | 25 | 25 |
| ART 7m-12m | 50.7 | 65 | 65 |
| ART > 12m | 64 | 75 | 75 |

**Table E. Model parameters.** Parameter values used in the model for each province. Where these differ between provinces they were adjusted (within the predefined ranges in the TIME software) to improve the manual fit. Full details of the parameter ranges used in TIME can be found in [[7](#_ENREF_7)].

**S.3. Additional Results**


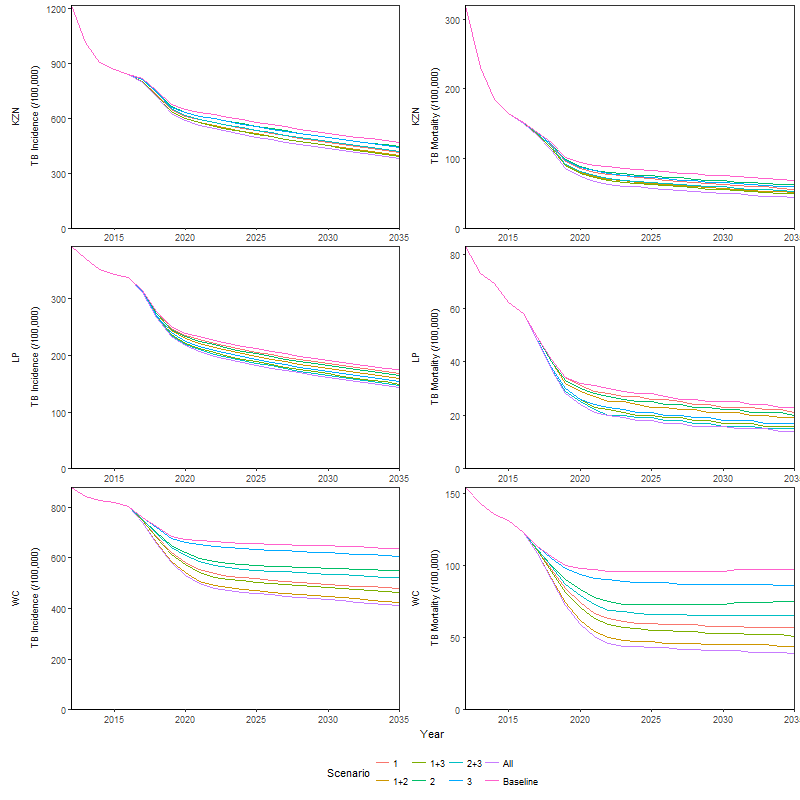


**Figures B.** Trends in TB incidence and mortality predicted by the model for each of the intervention scenarios on from 2012 to 2035 for each province.

**References**

1. Department of Health, R.o.S.A., *National tuberculosis management guidelines*. 2014: Pretoria, South Africa.

2. World Health Organisation, *Systematic screening for active tuberculosis. Principles and recommendations*. 2013, Geneva: WHO.

3. Steingart, K.R., et al., *Xpert(R) MTB/RIF assay for pulmonary tuberculosis and rifampicin resistance in adults.* Cochrane Database Syst Rev, 2013. **1**: p. CD009593.

4. Theron, G., et al., *Feasibility, accuracy, and clinical effect of point-of-care Xpert MTB/RIF testing for tuberculosis in primary-care settings in Africa: a multicentre, randomised, controlled trial.* Lancet, 2014. **383**(9915): p. 424-35.

5. MacPherson, P., et al., *Pre-treatment loss to follow-up in tuberculosis patients in low- and lower-middle-income countries and high-burden countries: a systematic review and meta-analysis.* Bull World Health Organ, 2014. **92**(2): p. 126-38.

6. Churchyard, G.J., et al., *Xpert MTB/RIF versus sputum microscopy as the initial diagnostic test for tuberculosis: a cluster-randomised trial embedded in South African roll-out of Xpert MTB/RIF.* Lancet Glob Health, 2015. **3**(8): p. e450-7.

7. Houben, R.M., et al., *TIME Impact - a new user-friendly tuberculosis (TB) model to inform TB policy decisions.* BMC Med, 2016. **14**: p. 56.
